# Whole genome sequencing identifies zoonotic transmission of MRSA isolates with the novel *mecA* homologue *mecC*

**DOI:** 10.1002/emmm.201202413

**Published:** 2013-03-25

**Authors:** Ewan M Harrison, Gavin K Paterson, Matthew TG Holden, Jesper Larsen, Marc Stegger, Anders Rhod Larsen, Andreas Petersen, Robert L Skov, Judit Marta Christensen, Anne Bak Zeuthen, Ole Heltberg, Simon R Harris, Ruth N Zadoks, Julian Parkhill, Sharon J Peacock, Mark A Holmes

**Affiliations:** 1 Department of Veterinary Medicine, University of CambridgeCambridge, UK; 2 Wellcome Trust Sanger InstituteHinxton, UK; 3 Microbiology and Infection Control, Statens Serum InstitutCopenhagen, Denmark; 4 Department of Clinical MicrobiologySlagelse Sygehus, Denmark; 5 Moredun Research InstitutePenicuik, UK; 6 Department of Clinical Medicine, University of CambridgeCambridge, UK

**Keywords:** cattle, *mecC*, MRSA, sheep, zoonosis

## Abstract

Several methicillin-resistant *Staphylococcus aureus* (MRSA) lineages that carry a novel *mecA* homologue (*mecC*) have recently been described in livestock and humans. In Denmark, two independent human cases of *mecC*-MRSA infection have been linked to a livestock reservoir. We investigated the molecular epidemiology of the associated MRSA isolates using whole genome sequencing (WGS). Single nucleotide polymorphisms (SNP) were defined and compared to a reference genome to place the isolates into a phylogenetic context. Phylogenetic analysis revealed two distinct farm-specific clusters comprising isolates from the human case and their own livestock, whereas human and animal isolates from the same farm only differed by a small number of SNPs, which supports the likelihood of zoonotic transmission. Further analyses identified a number of genes and mutations that may be associated with host interaction and virulence. This study demonstrates that *mecC*-MRSA ST130 isolates are capable of transmission between animals and humans, and underscores the potential of WGS in epidemiological investigations and source tracking of bacterial infections.

## INTRODUCTION

Methicillin-resistant *Staphylococcus aureus* (MRSA) causes a wide range of infections including skin and soft tissue infections, bacteremia, pneumonia and endocarditis. Molecular epidemiology has identified that a small number of multilocus sequence types (MLST) predominate in different regions of the world, including sequence type (ST) 239 in Asia and South America, and ST8 in the USA (Harris et al, 2010; Otto, 2012). MRSA infections are classified as healthcare-associated (HA-MRSA), community-acquired MRSA (CA-MRSA) and livestock-associated (LA-MRSA), which are often associated with certain STs and virulence factors (Chambers & Deleo, 2009; Fitzgerald, 2012). The potential for zoonotic transmission of *S. aureus* between livestock, companion animals and humans (Loeffler et al, 2011; Lowder et al, 2009; Pantosti, 2012) has been exemplified by the emergence of MRSA ST398 (Price et al, 2012). The link between humans and livestock have been further expanded by the recent description of MRSA isolates from the United Kingdom, Ireland and Denmark that harbour a divergent *mecA* homologue termed *mecC* (formerly *mecA*_LGA251_; Garcia-Alvarez et al, 2011; Ito et al, 2012; Shore et al, 2011). The penicillin-binding protein encoded by *mecC* differs from that of *mecA*, in having a higher relative affinity for oxacillin as compared to cefoxitin and has a different temperature sensitivity (Kim et al, 2012). *mecC-*positive MRSA isolates (*mecC*-MRSA) pose a potential public health problem, as the degree of nucleotide divergence between *mecC* and *mecA* means that they are negative when using current diagnostic tests such as PCR assays and latex agglutination tests that detect *mecA* and PBP2a, respectively (Stegger et al, 2012). The initial discovery of *mecC*-MRSA in the United Kingdom revealed that isolates from cattle and humans in geographic proximity were highly related, suggesting transmission between the two (Garcia-Alvarez et al, 2011). More recent work has identified that *mecC* is present in isolates from a range of STs and clonal complexes (CCs) found in humans and a diverse range of animal species throughout Europe (Cuny et al, 2011; Laurent et al, 2012; Paterson et al, 2012). The incidence of *mecC*-MRSA in Denmark has increased between 2003 and 2011 (Petersen et al, 2013), which highlights the need for a better understanding of its clinical and molecular epidemiology.

In Denmark, two human cases of *mecC*-MRSA bacteremia and wound infection, respectively, have been linked to a livestock reservoir on the patients' farms (Petersen et al, 2013). However, isolates from the two farm clusters were indistinguishable by multilocus sequence typing (MLST) (ST130), *spa* typing (t843), pulsed-field gel electrophoresis (PFGE) and multilocus variable number tandem repeat analysis (MLVA) (MT429). Here, we investigated the molecular epidemiology of these isolates using whole genome sequencing (WGS). Phylogenetic analysis revealed two distinct farm-specific clusters comprising isolates from both the human case and their own livestock, whereas human and animal isolates from the same farm only differed by a small number of single nucleotide polymorphisms (SNPs), which supports zoonotic transmission. Our findings further highlight the role of livestock as a MRSA reservoir for human infection, and demonstrates the power of WGS in epidemiological investigations and source tracking.

## RESULTS

### Investigation of zoonotic transmission

The first case was a 53-year-old female (Patient A) with a blood culture isolate (A1) and a nasal swab isolate (A2) positive for *mecC-*MRSA. The second patient was a 69-year-old female (Patient B) with a wound infected with *mecC*-MRSA (Patient B). Patient A lived on a farm with two cows, two horses and a dog, while Patient B lived on a farm with a flock of ten sheeps. The two farms were located 45 km (28 miles) apart. Swabbing of the animals on Patient A's farm 13.5 months after the initial infection produced a *mecC-*MRSA isolate from one of the cows (Cow A). Swabbing on Patient B's farm produced *mecC*-MRSA isolates from three sheep (Sheep B1, B2 and B3), 6.5 months after the initial infection in the farmer. All human and livestock isolates from both farms had identical MLST types (ST130), *spa* types (t843),fingerprints (MLVA and PFGE), and antimicrobial resistance profiles (resistant to cefoxitin and penicillin but susceptible to all non-β-lactam antibiotics tested; Petersen et al, 2013).

**Figure 1:**
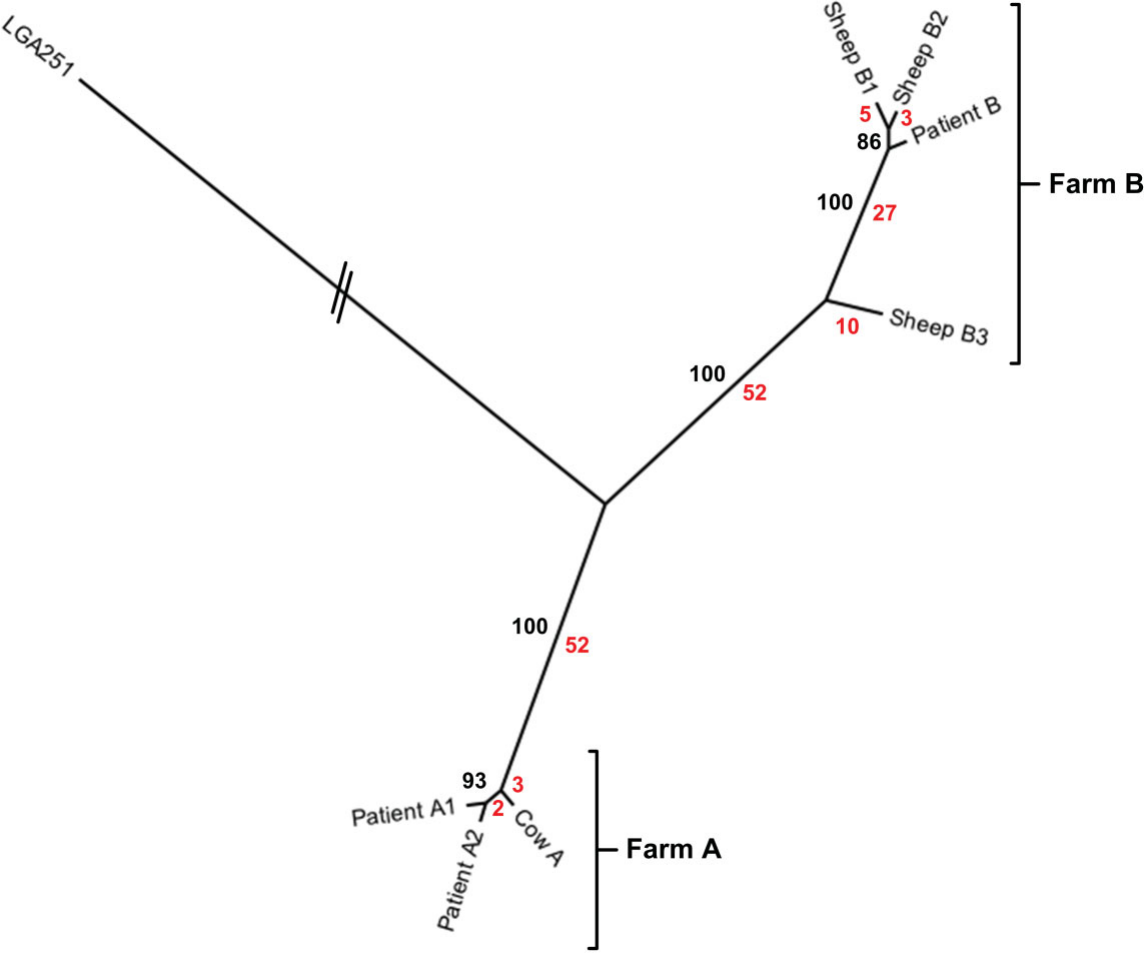
Phylogenetic relationships between human and animal isolates. Figure shows an unrooted maximum likelihood tree generated from SNPs in the core genome. The branch length for LGA251 has been trimmed. Bootstrap values for branches are shown in black. The number of differentiating SNPs for each branch is shown in red.

To elucidate if the two patients were infected by isolates from their livestock, we subjected the isolates to WGS. Phylogenetic analysis using SNPs in the core genome revealed that all the isolates were closely related, differing by a total of 154 SNPs (Fig. 1). The phylogeny also showed that human and livestock isolates from the same farm clustered into two separate clades which were differentiated by 104 SNPs (Fig. 1); Farm A (isolates from Cow A and Patient A [Patient A1 and Patient A2]) and Farm B (isolates from Sheep B1, B2, B3 and Patient B). For Patient A, the isolate from the nasal swab (Patient A1) and the isolate from the blood culture (Patient A2) have no differentiating SNPs, consistent with the farmer being colonized prior to invasive infection. The isolate from the cow on patient A's farm differed from the patient's two isolates by a total of five SNPs, suggesting that a transmission event had occurred although the direction of transmission remains unclear. For Patient B, the two isolates from Sheep B1 and B2 were most closely related to the farmer's isolate, differing by five and three different SNPs, respectively. A third isolate from Sheep B3 differed from Sheep B2 and Sheep B1 by forty and forty-two SNPs, respectively, and from the farmer's isolate by thirty-seven SNPs. This level of diversity between sheep isolates suggests that *mecC*-MRSA ST130 may have been circulating in the flock for an extended undefined period of time, or had been introduced into the flock on at least two occasions prior to the human infection in order to allow this number of SNPs to accumulate. Thus, in the case patient B, it is most likely that the direction of transmission was from sheep to human.

### Accessory genome and virulence factors

We further assessed the level of diversity between the isolates by interrogating the mobile gene content by comparative genomics. The mobile gene content of the strains in the two clades was similar, except that all isolates in the Farm A clade contained a ∼25-kb phage that is present in the bovine *mecC*-MRSA ST425 reference strain LGA251 (Garcia-Alvarez et al, 2011). The isolate from Sheep B3 lacked a φSa8 phage that was present in all of the other isolates in the study. To further understand the propensity of the *mecC*-MRSA ST130 isolates to cause disease in both animals and humans we assessed the genome sequences for *S. aureus* virulence factors. None of the isolates were positive for the genes encoding Panton-Valentine leucocidin (*lukS-PV* and *lukF-PV*) or the φSa3 phage-encoded modulators of the human innate immune responses, including SAK (*sak*), CHIPS (*chp*), and SCIN (*scn*). However, all isolates were positive for the genes encoding α-(*hla*), β-(*hlb*) and γ-haemolysin (*hlgACB*), leucocidin ED (*lukED*), exfoliative toxin A (*eta*), exfoliative toxin B (*etb*), epidermal cell differentiation inhibitor-B (*edin-B*), and *set2*, *set3*, *set4*, *set5*, *set7*, *set10*. All isolates carried a novel allele of *etd* that shared only 59% nucleotide identity with the previously described *etd* gene (Nishifuji et al, 2008). Furthermore, all isolates were positive for a putative uncharacterised enterotoxin also present in the LA-MRSA ST398 reference strain S0385 (SAPIG1666). All isolates also had an intact SCC*mec* type XI.

### SNP and Indel analysis of ST130 isolates

We analysed the locations of SNPs that differentiated the isolates in the phylogenetic analysis (Fig. 1) to identify any SNPs that were potentially associated with host interaction (Supporting Information Tables 3 and 6). Of the 154 SNPs that differentiated the isolates, 84 (54.5%) were non-synonymous (of which 3 (1.9%) were stop codons), 25 (16.2%) were synonymous, and 45 were intergenic (29.2%), similar values to those reported in a larger study of ST239 isolates (Castillo-Ramirez et al, 2011). Fourteen genes, eleven of which were present in all isolates, contained SNPs causing premature stop codons, seven of which would be predicted produce significantly truncated protein products (Table 1). Of interest, the four strains in the Farm B clade had a premature stop-codon, Ile369Stop in *sbi* (SARLGA251_22000), encoding a protein involved immunoglobulin binding and evasion of the complement system (Haupt et al, 2008; Table 1). Another stop codon was present in all strains, 20 bp from the 3′-end of *rlmM* (SARLGA251_11290), encoding a ribosomal RNA large subunit methyltransferase (Table 1). Finally, we identified two deletions in genes associated with virulence and antibiotic resistance (Supporting Information Table 5). Firstly, all the isolates had a ∼3.3 kb deletion of the collagen adhesin-encoding gene (*cna*), a virulence factor implicated in the pathogenesis of a range of infection types (Elasri et al, 2002; Rhem et al, 2000). Secondly, all isolates have a ∼2.3 kb deletion of the region encoding an EamA-like transporter family protein (SALGA251_00820) and *norG* (SALGA251_00830), a global regulator of multidrug resistance (MDR) transporters and efflux pumps (Truong-Bolduc et al, 2011).

**Table 1:** SNPs causing premature stop codons in the ST130 isolates in this study

LGA251 locus	gene	LGA251 annotated function	position in CDS^a^ (bp)	CDS size (bp)	Ref Base^b^	SNP base	Farm A			Farm B			
	
							Cow A	Patient A1	Patient A2	Sheep B1	SheepB2	Sheep B3	Patient B
SARLGA251_01530		Putative amino acid kinase	763	770	A	T	Yes	Yes	Yes	Yes	Yes	Yes	Yes
SARLGA251_03380		Putative membrane protein	238	383	C	T	Yes	Yes	Yes	Yes	Yes	Yes	Yes
SARLGA251_03400		Hypothetical protein	129	221	G	A	Yes	Yes	Yes	Yes	Yes	Yes	Yes
SARLGA251_03890		Putative membrane protein	129	416	T	A	Yes	Yes	Yes	Yes	Yes	Yes	Yes
SARLGA251_04910		Haloaciddehalogenase-like hydrolase	682	713	C	A	Yes	Yes	Yes	Yes	Yes	Yes	Yes
SARLGA251_11290	*rlmN*	Ribosomal RNA large subunit methyltransferase N	1074	1094	C	T	Yes	Yes	Yes	Yes	Yes	Yes	Yes
SARLGA251_12160		HflX GTP-binding protein	1146	1238	C	T	Yes	Yes	Yes	No	No	No	No
SARLGA251_15620	*hemD*	Uroporphyrinogen III synthase	666	674	T	A	Yes	Yes	Yes	Yes	Yes	Yes	Yes
SARLGA251_18510		Putative membrane protein	236	1610	G	A	Yes	Yes	Yes	Yes	Yes	Yes	Yes
SARLGA251_18940	*thiM*	Putative hydroxyethylthiazole kinase	786	791	C	A	Yes	Yes	Yes	Yes	Yes	Yes	Yes
SARLGA251_19330		ATP-grasp domain containing protein	585	1193	G	A	Yes	Yes	Yes	Yes	Yes	Yes	Yes
SARLGA251_19760		Aerobactin biosynthesis protein	868	1976	G	A	No	No	No	No	No	Yes	No
SARLGA251_22000	*sbi*	IgG-binding protein	1104	1310	C	T	No	No	No	Yes	Yes	Yes	Yes
SARLGA251_22130		Putative glycerate kinase	1137	1142	C	T	Yes	Yes	Yes	Yes	Yes	Yes	Yes

a The location from the start codon in base pairs.

b Base in the reference genome LGA251.

## DISCUSSION

In this study, we have presented evidence of the zoonotic transmission of *mecC*-MRSA ST130 in two separate cases by the use of whole-genome sequencing. Although the SNP data alone does not provide clear proof for the direction of transmission, this data combined with the epidemiological evidence that CC130 isolates are commonly isolated from animals but rarely in humans, supports the case for zoonotic transmission (Jorgensen et al, 2005; Paterson et al, 2012). Due to the retrospective nature of this study we were unable to sequence multiple isolates from the same host to assess the ‘cloud of variation’ (the extent of genetic heterogeneity) present in the population colonizing each host (Harris et al, 2013; Young et al, 2012). Future studies of zoonotic transmission should include denser sampling of multiple isolates from each host to investigate this question. The finding that highly related isolates of ST130 are able to colonize three different species and that CC130 isolates have been isolated from a broad range of animal species (Paterson et al, 2012), suggests that CC130 might be better regarded as a generalist lineage rather than a ‘livestock’ specific sequence type as has been suggested for CC97 (Guinane et al, 2010). Further studies are required to investigate how commonly animal to human transmission events of *mecC*-MRSA CC130 isolates occur, and if long-term carriage persists in the community.

Analysis of the whole genome sequence of the study isolates identified that they lacked classical virulence factors such a PVL or the φSa3 phage-encoded virulence factors that are present in human associated ST398 isolates and other community associated MRSA lineages (Chambers & Deleo, 2009; Price et al, 2012). This could suggest that CC130 isolates have the ability to cause invasive disease in humans using other virulence factors. The recent finding that leucocidin ED specifically targets neutrophils and is an important virulence determinate in a murine bloodstream model makes this a likely candidate to play a key role in CC130 pathogenicity (Alonzo et al, 2012). In addition, the presence of three exfoliative toxins and an epidermal cell differentiation inhibitor (EDIN-A) may provide CC130 isolates with the ability to colonise a broad range of host species. In particular, the divergent ETD warrants further study as this may represent a host-adapted form, as staphylococcal exfoliative toxins are reported to exhibit tissue specificity to different animal species (Elias et al, 1976; Nishifuji et al, 2008).

We also identified a number of deletions in genes previously associated with host specificity, virulence and antibiotic resistance. The finding that all the isolates in the sheep associated clade (Farm B) contains an inactivated copy of *sbi,* a complement- and IgG-binding protein, may indicate host-specific gene decay, as Sbi is not able to bind sheep IgG (Atkins et al, 2008). We also identified a common deletion of *norG*, a global regulator involved in resistance to β-lactams (Truong-Bolduc et al, 2011). One previous study has investigated genetic changes in the progression from carriage to disease in *S. aureus*, in which a number of mutations were identified including a premature stop codon in an *AraC* family transcriptional regulator (AFTR) which potentially altered the virulence of the invasive isolate (Young et al, 2012). The two isolates in our study from carriage and the bloodstream of Patient A same patient were identical, confirming the importance of autoinfection in bacteremia and that the isolates were capable of causing severe disease in the absence of any genetic change. Given the restricted number of isolates presented in this study, it is not possible to draw any clear conclusions from the SNP/Indel data without further experimental characterization. Larger scale studies of ST130 isolates from zoonotic transmission and from carriage to disease transition are required to remove the stochastic noise from SNP accumulations which purifying selection has not had time to act upon (Castillo-Ramirez et al, 2011). Experimental investigations focusing on the role of individual virulence factors in the pathogenesis of CC130 isolates in different hosts should also be undertaken to help understand the basis of the broad host specificity of this lineage.

Finally, given the animal-to-human spread of LA-MRSA CC398 (Price et al, 2012) and now potentially CC130, surveillance of *S. aureus* and other animal pathogens from livestock and wildlife should be undertaken to monitor the emergence of new clones, and to further improve our understanding of bacterial pathogen evolution. In this study, we have described the first use of WGS to confirm animal-to-human transmission of *mecC*-MRSA isolates and to track a potentially emerging clone, further demonstrating the great value of rapid WGS as a tool in clinical epidemiology and source tracking, as has previously been demonstrated in the hospital setting (Koser et al, 2012).

**The paper explained**

**PROBLEM**

The emergence of livestock-associated methicillin-resistant *Staphylococcus aureus* (LA-MRSA) is a major public health concern. Recently, MRSA strains with a novel *mecA* homologue (*mecC*), which may go undetected by current diagnostic tests, were described in both livestock and humans suggesting potential zoonotic transmission. Denmark has reported a significant increase in cases of CC130 *mecC*-MRSA between 2003 and 2011 and two independent human cases of *mecC*-MRSA infection directly linked to a livestock reservoir have been identified.

**RESULTS**

We investigated the molecular epidemiology of these livestock-associated *mecC*-MRSA cases using WGS. Phylogenetic analysis across the entire core genome revealed that the isolates from these cases form two distinct, farm-specific clusters comprising near identical isolates from the human case and from livestock on that farm. Within each cluster, the human and animal isolates only differed by a small number of SNPs, which supports the premise of zoonotic transmission. In-depth genome analysis identified a number of candidate genes and mutations that may be associated with host–pathogen interactions and virulence of this emerging MRSA clone.

**IMPACT**

Our findings demonstrate that the CC130 MRSA lineage is capable of transmission between animals and humans, further highlighting the role of livestock as a reservoir for MRSA. Our study also underscores the potential of WGS in epidemiological investigations and source tracking of bacterial infections.

## MATERIALS AND METHODS

### Clinical information

Patient A was a 53-year-old female who had been treated with steroid injections in the shoulder and hip for a degenerative condition. A subcutaneous abscess appeared on the shoulder 9 days after injection. The patient was admitted to hospital 2 weeks post-injection. She was febrile and complained of pain in her shoulder and back. She was initially treated with IV ampicillin and gentamicin. After 24 h, treatment was changed to IV cefuroxime. Severe pain and fever persisted, and blood CRP (C-reactive protein) showed a minor decrease from 345 to 219 mg/l (ref < 8) during the first 3 days of treatment. A CT-scan suggested an extradural spinal abscess at the T10 level, extending distally. Blood cultures from the day of admission grew a MRSA. Treatment was successfully completed with clindamycin 600 mg TID IV, rifampicin 300 mg BID, followed by fusidic acid 500 mg TID and rifampicin 300 mg BID for 6 weeks. Patient B was a 69-year-old female with a chain saw wound that severed several muscles and arteries in the forearm and required reconstructive surgery in an orthopedic referral centre. 10 days after the surgery she presented at the out-patient clinic with a low grade wound infection. She received empirical treatment with oral penicillin and di-cloxacillin. A MRSA strain was isolated from the wound but specific antibiotic treatment was considered unnecessary, and the wound healed.

### Whole genome sequencing

Genomic DNA was extracted from overnight cultures grown in TSB using the MasterPure Gram Positive DNA Purification Kit, Cambio, UK. Illumina library preparation was carried out as described by Quail et al. (Quail et al, 2008), and Hi-seq sequencing was carried out following the manufacturer's standard protocols (Illumina, Inc, USA). Nucleotide sequences of isolates from Patient A1, Patient A2, Cow A, Patient B and Sheep B1, B2 and B3, have been deposited in the European short read archive with accession numbers: ERR084771, ERR084772, ERR144792, ERR144788, ERR144771, ERR144772 and ERR144749, respectively.

### Phylogenetics and comparative genomics

Fastq files for the isolates were mapped against the *mecC*-MRSA reference genome LGA251 (EMBL accession no: FR821779) using SMALT (http://www.sanger.ac.uk/smalt) in order to identify SNPs, as previously described (Koser et al, 2012). SNPs located in mobile genetic elements (Supporting Information Table 1) or low quality regions (insertions and deletions (Indels)/low coverage/repeat regions) (Supporting Information Table 2) were identified by manual inspection and removed from the alignment. The maximum likelihood tree was generated from the resulting SNPs present in the core genome (the core genome being defined as the regions of the chromosome not excluded when all Indel and mobile genetic elements were removed) usingRAxML (Stamatakis et al, 2005). Insertions and deletions (indels) were identified as previously described (Croucher et al, 2011). Indels of potential biological interest were manually accessed using BAM files mapped on the reference. Comparison of the mobile genetic content of the isolates was assessed by BLAST analysis against Velvet *de novo* assemblies using known *S. aureus* mobile elements and phage integrases downloaded from EMBL and NCBI databases (Zerbino & Birney, 2008). Comparative genomics were carried out using Velvet *de novo* assemblies with contigs realigned using Mauve (Darling et al, 2004) and manually using Artemis comparison tool (Carver et al, 2005). *S. aureus* virulence factors from the literature were identified using BLAST against Velvet assemblies.

## Author contributions

EMH designed and performed analyses, interpreted data and wrote the manuscript. GKP, MTGH, JL and MS performed analyses and interpreted data and contributed to the manuscript. JMC, ABZ and OH conducted epidemiological follow up and isolated the strains. ARL, AP and RLS performed experiments, interpreted data and contributed to the manuscript. SRH provided analytical tools. RNZ, JP and SJP interpreted data and contributed to the manuscript. MAH initiated and designed the study, interpreted data and wrote the manuscript.

## Notes

As a service to our authors and readers, this journal provides supporting information supplied by the authors. Such materials are peer reviewed and may be re-organized for online delivery, but are not copy-edited or typeset. Technical support issues arising from supporting information (other than missing files) should be addressed to the authors.
