# Supplementary material for: Whole genome sequencing identifies zoonotic transmission of MRSA isolates with the novel mecA homologue mecC
Source: EMBO Mol Med. 2013 Mar 25;5(4):509–15. doi: 10.1002/emmm.201202413 (PMC3628104; doi:10.1002/emmm.201202413)
Supplement: Supplementary file 1 [file emmm0005-0509-sd1.pdf]

Whole genome sequencing identifies zoonotic transmission of MRSA isolates with the novel mecA homologue mecC

Ewan M. Harrison, Gavin K. Paterson, Matthew T.G. Holden, Jesper Larsen, Marc Stegger, Anders Rhod Larsen, Andreas Petersen, Robert L. Skov, Judit Marta Christensen, Anne Bak Zeuthen, Ole Heltberg, Simon R. Harris, Ruth N. Zadoks, Julian Parkhill, Sharon J. Peacock, Mark A. Holmes

Corresponding author: Mark Holmes, University of Cambridge

Review timeline:

Submission date:	21 December 2012
Editorial Decision:	29 January 2013
Revision received:	06 February 2013
Accepted:	08 February 2013

Transaction Report:

(Note: With the exception of the correction of typographical or spelling errors that could be a source of ambiguity, letters and reports are not edited. The original formatting of letters and referee reports may not be reflected in this compilation.)

Editor: Roberto Buccione

1st Editorial Decision 29 January 2013

Thank you for the submission of your manuscript to EMBO Molecular Medicine. We have now heard back from the three Reviewers whom we asked to evaluate your manuscript.

You will see that both Reviewers are supportive of your work and underline its considerable potential interest. Reviewers 1 and 3 have minor concerns and suggest some improvements that I would advise you to take action upon. Reviewer 2 instead feels that the section on putative virulence factors is too descriptive and insufficiently supported. I would encourage you to integrate the manuscript with further experimental support if available (or attainable in the very short term). In alternative, I would be prepared to accept a textually amended manuscript aimed at easing this concern.

In conclusion, although publication of the paper cannot be considered at this stage, we would be pleased to consider a suitably revised version.

Please note that it is EMBO Molecular Medicine policy to allow a single round of revision only and that, therefore, acceptance or rejection of the manuscript will depend on the completeness of your responses included in the next, final version of the manuscript.

I look forward to reading a new revised version of your manuscript as soon as possible.

***** Reviewer's comments *****

Referee #1 (General Remarks):

The work entitled "Whole genome sequencing identifies zoonotic transmission of MRSA isolates with the novel mecA homologue mecC" by E Harrisonet al. describes the phylogenetic characteristics and relationships of mecC carrying MRSA. It demonstrates very nice how whole genome analyses give the necessary detail information to unravel the epidemiology of bacterial infections where traditional molecular typing methods fail.
One comment: the authors use the terminology of core- and accessory genome but it is not clear what the sets of genes they contain. It seems that they use the chromosomal genome as core and the mobile gene content as accessory. But also the chromosomal genome may contain accessory genes. If they calculated the core genome from the set of chromosomal genomes the method should be described.

Referee #2 (General Remarks):

Harisson et al. investigated the molecular epidemiology of livestock-associated (CC)130 mecC-MRSA using a whole genome sequencing approach. Based on phylogenetic analysis, the authors provided evidence of farm-specific clusters supporting the possibility of zoonotic transmission of the CC130 MRSA lineage. This is one of the first demonstrations of the use of whole genome sequencing of bacteria to demonstrate zoonotic transmission (although one cannot exclude transmission of man to animals). On that basis this is thus an interesting paper. However they are concerns also (see below).
1. The phylogeny part is fairly convincing and it is a great use of whole genome sequencing.
2. The search for putative virulence factors is very descriptive and not much in line with the main story of the paper (zoonotic transmission). The number of strains studied is too small to make any conclusions. Alternatively, if the number of strains cannot be increased, functional validation of some of the hypotheses would be required.

Referee #3 (General Remarks):

The submission by Harrison and colleagues describes an elegant combination of classical epidemiological investigation with the most modern molecular typing techniques in order to unravel what appears to be a zoonotic transmission of MRSA carrying the recently described mecC determinant. The observations were made at two farms and the carrier/components of the epidemiological scenario are a human and sheep in one of the cases and a human and cows in the other. The application of whole genome sequencing to the isolates clearly identifies the MRSA strains which are equipped with the recently described mecC determinant. It is interesting that in order to provide a convincing argument for the direction of the transmission the authors had to combine the high resolution SNP data derived from full sequencing of all the isolates with the more old fashioned empirical evidence which suggests that the direction of transmission was from animals to the humans. The full genome sequencing allowed the authors to clearly identify the components of these two "outbreaks" as two distinct MRSA clusters which were specific for the particular farm.

A special compliment is due to the authors for writing a manuscript which is easily readable for a more general readership not necessarily fully familiar with the language of sequencing and molecular genetics.
The paper abounds in an extremely interesting catalogue of observations which illustrate how an intelligent use of full genome sequencing can actually provide guidance in resolving epidemiological puzzles. Even beyond providing a convincing scenario for the direction of transmission that occurred at these two farms, the paper also examines other issues of basic significance. For instance, nasal and blood culture isolates are compared by whole genome sequencing and are shown to have no clear evidence of a different set of SNPs suggesting that the farmers were first colonized with the bacteria and then became ill with the disease.

The availability of these isolates allowed the authors to provide an interesting catalogue of virulence

factors carried by these newly identified MRSA clones. This catalogue includes the number and nature of the different virulence factors and outstanding is the absence of the pvl toxin or phage associated virulence factors that are often seen in the most frequent MRSA clones. The authors actually proceed to search for alternative virulence factors that may be associated with the spread and disease causing potential of these new MRSA clones.

One bit of information that I would suggest that the authors add to their manuscript is the nature of the cassette chromosome carried by the isolates which - I assume is the SCCXI that were shown to be unique to isolates carrying the mecC determinant.
In summary, I consider this an elegant demonstration of how to use the latest molecular biological tools to the analysis of transmission of pathogenic bacteria between hosts that include animals and humans. The manuscript also serves as an example in lucidity of style which makes the content of the paper accessible for readers of very different preparation in epidemiology.

1st Revision - authors' response 06 February 2013

Referee #1 (General Remarks):

The work entitled "Whole genome sequencing identifies zoonotic transmission of MRSA isolates with the novel mecA homologue mecC" by E Harrison et al. describes the phylogenetic characteristics and relationships of mecC carrying MRSA. It demonstrates very nice how whole genome analyses give the necessary detail information to unravel the epidemiology of bacterial infections where traditional molecular typing methods fail.

One comment: the authors use the terminology of core- and accessory genome but it is not clear what the sets of genes they contain. It seems that they use the chromosomal genome as core and the mobile gene content as accessory. But also the chromosomal genome may contain accessory genes. If they calculated the core genome from the set of chromosomal genomes the method should be described.

Response: We thank the reviewer for their supportive comments. In regard to the terminology of core- and accessory genome, we have now added the following statement to clarify the issue: 'The maximum likelihood tree was generated from the resulting SNPs present in the core genome (the core genome being defined as the regions of the chromosome not excluded when all Indel and mobile genetic elements were removed) using RAxML (Stamatakis et al, 2005).' (Lines: 249 to 252).

Referee #2 (General Remarks):

Harisson et al. investigated the molecular epidemiology of livestock-associated (CC)130 mecC-MRSA using a whole genome sequencing approach. Based on phylogenetic analysis, the authors provided evidence of farm-specific clusters supporting the possibility of zoonotic transmission of the CC130 MRSA lineage. This is one of the first demonstrations of the use of whole genome sequencing of bacteria to demonstrate zoonotic transmission (although one cannot exclude transmission of man to animals). On that basis this is thus an interesting paper. However they are concerns also (see below).

1. The phylogeny part is fairly convincing and it is a great use of whole genome sequencing.

2. The search for putative virulence factors is very descriptive and not much in line with the main story of the paper (zoonotic transmission). The number of strains studied is too small to make any conclusions. Alternatively, if the number of strains cannot be increased, functional validation of some of the hypotheses would be required.

Response: We thank the reviewer for their helpful comments. In response to point 2, we accept that the section on virulence factors is based on a limited number of strains and not backed up by experimental data. To generate and characterize mutants is a significant undertaking beyond the scope of the current investigation to use genome sequencing to examine zoonotic transmission. We do not have the resources to undertake this work and we note that CC130 strains have only been superficially characterized and that no molecular biology tools or phenotyping assays have been applied to this lineage. This would add further to the time required to undertake such work. However, we understand the reviewers concerns, so we have reduced the data regarding SNPs potentially implicated in virulence to only those describing premature stop codons and large-scale deletions and have now moved Table 2 to the supplementary material (Table S6). We would argue that the remaining data and analysis of potential virulence factors is pertinent as this is the first publication describing the genomes of ST130 isolates from human infections. In particular, as CC130 is an emerging multi-host lineage of MRSA that lacks both PVL or other 'classic' *S. aureus* human virulence factors associated with community / livestock associated MRSA, analysis of potential determinates of virulence should be of interest to a broad readership (as highlighted by Reviewer 3). We would also argue that the analysis of deletions and SNPs present in the genomes fits with the focus on zoonotic transmission, as this analysis is focused on highlighting putative adaptations that enable these strains to move between both animals and humans. We have tried to be as clear as possible about the caveats of our current analysis in the discussion and that these are putative annotations without experimental validation and the associations are seen in a limited number of strains: 'Given the restricted number of isolates presented in this study, it is not possible to draw any clear conclusions from the SNP / Indel data without further experimental characterization. Larger scale studies of ST130 isolates from zoonotic transmission and from carriage to disease transition are required to remove the stochastic noise from SNP accumulations which purifying selection has not had time to act upon (Castillo-Ramirez et al, 2011)'. (Lines: 197 to 202). Analysis of more sequenced strains and functional work are absolutely needed to fully understand CC130 disease pathogenesis and host adaption events, but we would strongly argue that the value of the current analysis and discussion is to highlight candidate genes for future investigation by the wider research community. We have now added additional comment to the discussion to highlight this: 'Experimental investigations focusing on the role of individual virulence factors in the pathogenesis of CC130 isolates in different hosts should also be undertaken to help understand the basis of the broad host specificity of this lineage.' (Lines: 202-204)

Referee #3 (General Remarks):

The submission by Harrison and colleagues describes an elegant combination of classical epidemiological investigation with the most modern molecular typing techniques in order to unravel what appears to be a zoonotic transmission of MRSA carrying the recently described mecC determinant. The observations were made at two farms and the carrier/components of the epidemiological scenario are a human and sheep in one of the cases and a human and cows in the other. The application of whole genome sequencing to the isolates clearly identifies the MRSA strains which are equipped with the recently described mecC determinant. It is interesting that in order to provide a convincing argument for the direction of the transmission the authors had to combine the high resolution SNP data derived from full sequencing of all the isolates with the more old fashioned empirical evidence which suggests that the direction of transmission was from animals to the humans. The full genome sequencing allowed the authors to clearly identify the components of these two "outbreaks" as two distinct MRSA clusters which were specific for the particular farm.

A special compliment is due to the authors for writing a manuscript which is easily readable for a more general readership not necessarily fully familiar with the language of sequencing and molecular genetics.

The paper abounds in an extremely interesting catalogue of observations which illustrate how an intelligent use of full genome sequencing can actually provide guidance in resolving

epidemiological puzzles. Even beyond providing a convincing scenario for the direction of transmission that occurred at these two farms, the paper also examines other issues of basic significance. For instance, nasal and blood culture isolates are compared by whole genome sequencing and are shown to have no clear evidence of a different set of SNPs suggesting that the farmers were first colonized with the bacteria and then became ill with the disease.

The availability of these isolates allowed the authors to provide an interesting catalogue of virulence factors carried by these newly identified MRSA clones. This catalogue includes the number and nature of the different virulence factors and outstanding is the absence of the pvl toxin or phage associated virulence factors that are often seen in the most frequent MRSA clones. The authors actually proceed to search for alternative virulence factors that may be associated with the spread and disease causing potential of these new MRSA clones.

One bit of information that I would suggest that the authors add to their manuscript is the nature of the cassette chromosome carried by the isolates which - I assume is the SCCXI that were shown to be unique to isolates carrying the mecC determinant.

In summary, I consider this an elegant demonstration of how to use the latest molecular biological tools to the analysis of transmission of pathogenic bacteria between hosts that include animals and humans. The manuscript also serves as an example in lucidity of style which makes the content of the paper accessible for readers of very different preparation in epidemiology.

Response: We thank the reviewer for their positive comments. We have now clarified that all the isolates harbour an intact SCC*mec* type XI" 'All isolates also had an intact SCC*mec* type XI' (see line: 131).

© EMBO
